# Cadherins and catenins in cancer: connecting cancer pathways and tumor microenvironment

**DOI:** 10.3389/fcell.2023.1137013

**Published:** 2023-05-15

**Authors:** Wan-Hsin Lin, Lisa M. Cooper, Panos Z. Anastasiadis

**Affiliations:** Department of Cancer Biology, Mayo Clinic, Jacksonville, FL, United States

**Keywords:** cadherins, Yap/Taz, growth factor receptors, WNT, Notch, hedgehog, epithelial-mesenchymal transition, immune responses

## Abstract

Cadherin-catenin complexes are integral components of the adherens junctions crucial for cell-cell adhesion and tissue homeostasis. Dysregulation of these complexes is linked to cancer development via alteration of cell-autonomous oncogenic signaling pathways and extrinsic tumor microenvironment. Advances in multiomics have uncovered key signaling events in multiple cancer types, creating a need for a better understanding of the crosstalk between cadherin-catenin complexes and oncogenic pathways. In this review, we focus on the biological functions of classical cadherins and associated catenins, describe how their dysregulation influences major cancer pathways, and discuss feedback regulation mechanisms between cadherin complexes and cellular signaling. We discuss evidence of cross regulation in the following contexts: Hippo-Yap/Taz and receptor tyrosine kinase signaling, key pathways involved in cell proliferation and growth; Wnt, Notch, and hedgehog signaling, key developmental pathways involved in human cancer; as well as TGFβ and the epithelial-to-mesenchymal transition program, an important process for cancer cell plasticity. Moreover, we briefly explore the role of cadherins and catenins in mechanotransduction and the immune tumor microenvironment.

## Introduction

The integrity of the epithelial monolayer is critical for tissue morphogenesis and depends on dynamic interactions between cells to maintain tissue homeostasis. A group of transmembrane proteins termed cadherins and their associated catenins play a critical role in these interactions at areas of cell-cell contact. Cadherin-catenin complexes (CCC) coalesce to form adherens junctions (AJs), which in addition to their adhesive function, are dynamic structures and hubs for intracellular signaling conveying signals related to cohesion, tension, proliferation, and inflammation. The ultimate goal of this signaling is maintenance of epithelial barrier function and tissue homeostasis.

Not surprisingly, dysfunction of the CCC is associated with loss of epithelial architecture, increased cell proliferation and survival, as well as enhanced cell migration and invasion. In line with these altered cellular behaviors, CCC components are frequently deregulated in human cancer and implicated in cancer progression. For example, loss of epithelial cadherin (E-cadherin, encoded by *CDH1*) is considered as the most prominent cancer driving event in invasive lobular carcinoma of the breast (ILC) (Ciriello et al., 2015), as well as hereditary and a subset of sporadic diffuse gastric cancer (Wang et al., 2014; Cho et al., 2017). The mechanism by which E-cadherin loss promotes cancer has been studied in several animal models [reviewed in (Bruner and Derksen, 2018)]. Examples of catenin misregulation include recurrent homozygous deletion of α-catenin (encoded by *CTNNA1*) in basal-like breast cancer (Ding et al., 2010). Genomic alterations of p120-catenin (encoded by *CTNND1*) are relatively rare in human cancer (cBioPortal). Epigenetic downregulation of p120-catenin expression promotes E-cadherin degradation and cancer progression in non-small-cell lung cancer (Mortazavi et al., 2010). Conversely, high p120-catenin expression is thought to be a key event in the progression of inflammatory breast cancer (Silvera et al., 2009).

Advances in sequencing technologies and multi-omics approaches are unraveling the oncogenic landscape of human tumors, including the elucidation of key oncogenic signaling pathways that play major roles in tumor initiation and progression. In this review, after a brief overview of CCC organization and function, we discuss recent advances in our understanding of the relationship between cadherins/catenins and oncogenic pathways, including Hippo-YAP/TAZ, receptor tyrosine kinase (RTK), WNT, Notch, hedgehog (HH), and TGFβ/epithelial-mesenchymal transition (EMT) pathways. In each section, we cover the introduction of an oncogenic pathway of interest, its involvement in particular cancer types according to omics data, and its bidirectional interplay with cadherins and catenins. As most human cancer is epithelial in origin, our focus will be E-cadherin and its related catenins, with occasional discussion on roles of other classical cadherins, such as neural cadherin (N-cadherin, encoded by *CDH2*) and vascular cadherin (VE-cadherin, encoded by *CDH5*). The role of cadherins/catenins in mechanotransduction and their crosstalk with the tumor microenvironment (TME) will also be discussed.

### Cadherin-catenin complex

**FIGURE 1:**
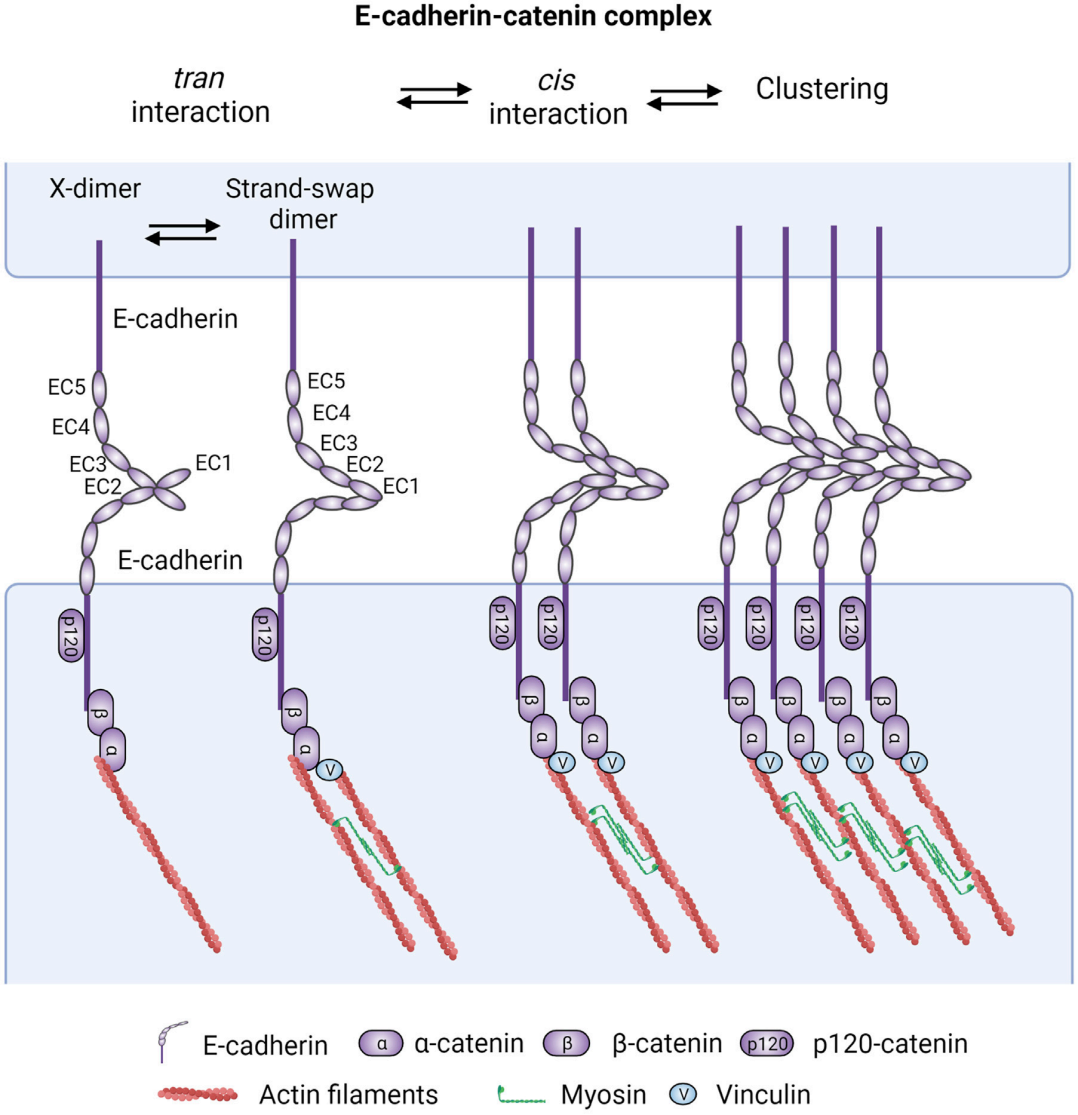
The core epithelial cadherin-catenin cell adhesion system. Epithelial cadherin-catenin complexes are the main building blocks of intercellular adhesions and are comprised of at least membrane-bound E-cadherin associated with p120-catenin (p120), β-catenin (β-cat), and α-catenin (α-cat). Fundamentally, these adhesions are mediated by homophilic E-cadherin adhesive (*trans* dimerization forming the X-dimer and strand-swap dimer) interactions between the ectodomains (EC) of cadherins on opposite cells and lateral (*cis*) binding between cadherin molecules on the same cell. These *cis*/*trans* interactions reciprocally strengthen each other’s stability, building up cadherin clusters. The clustering dynamics and stability can be further fine tuned by cellular and external biochemical or mechanical inputs. Of note, p120 binding to E-cadherin has also been shown to strengthen E-cadherin *cis* dimerization and *trans* interaction Additionally, intracellular actomyosin-mediated pulling forces, which are linked to cadherin molecules through α-catenin binding to β-catenin, further potentiate the stability of adherens junctions through a tension-mediated recruitment of vinculin (v) and its interaction with actin filaments. Figure created with BioRender.com.

E-cadherin is the prototypical member of the classical cadherin family. It is a transmembrane adhesion receptor that contains five extracellular repeats, a single-span transmembrane region, and a cytoplasmic tail (Figure 1). E-cadherin ectodomains (ECs) mediate calcium-dependent homophilic interactions with E-cadherins in the same (*cis* interactions via EC1 in one cadherin and EC2 and EC3 of an adjacent cadherin) and apposing cells (*trans* interactions via EC1 from both cadherin molecules from each cell, existing in weak X-dimer and strong strand-swap dimer conformations) (Yagi and Takeichi, 2000; Wu et al., 2010; Harrison et al., 2011; Wu et al., 2011; Brasch et al., 2012; Fichtner et al., 2014) that cooperatively strengthen each other’s stability (Zhang et al., 2009; Thompson et al., 2021). The structural and dynamic *cis*/*trans* interactions of E-cadherin molecules are beyond the scope of this review and have been reviewed in detail elsewhere (Brasch et al., 2012; Troyanovsky, 2022). On the other hand, the extracellular adhesive contact is further enforced by interaction of E-cadherin’s cytoplasmic tail with various catenins (inside-out signaling) (Nagafuchi and Takeichi, 1988; Ozawa et al., 1989; Thoreson et al., 2000; Gottardi and Gumbiner, 2001; Petrova et al., 2012; Shashikanth et al., 2015; Maiden et al., 2016; Koirala et al., 2021) (Figure 1).

It is noteworthy that cadherins associate with a large number of proteins that collectively form the cadherin adhesome (also known as cadhesome) and structurally support and/or regulate the dynamics of cadherin junctions (Guo et al., 2014; Van Itallie et al., 2014; Bertocchi et al., 2017; Li et al., 2019; Shafraz et al., 2020). These associations are thought to occur at distinct microclusters during the process of CCC clustering [reviewed in (Yap et al., 2015; Troyanovsky, 2022)]. While many of these interacting proteins are likely to have roles in cancer, this review will focus primarily on catenins, the most studied and better understood cadherin interacting partners that mediate linkage to the cytoskeleton and regulate adhesion-induced signaling. β-catenin (encoded by CTNNB1) binds directly to the C-terminal “catenin-binding domain” of the cadherin cytoplasmic tail (Nagafuchi and Takeichi, 1988; Ozawa et al., 1989; Ozawa et al., 1990; McCrea and Gumbiner, 1991; Stappert and Kemler, 1994; Thoreson et al., 2000; Gottardi and Gumbiner, 2001; Huber and Weis, 2001) and recruits alpha epithelial catenin (αE-catenin) to bridge E-cad adhesions to the actin cytoskeleton (Rimm et al., 1995; Drees et al., 2005; Desai et al., 2013). αE-Catenin also functions as allosteric regulator of AJ remodeling in a force-dependent manner, acting as a tension transducer via its homolog vinculin (Peng et al., 2012; Thomas et al., 2013; Yao et al., 2014; Ishiyama et al., 2018; Seddiki et al., 2018). Actomyosin tension changes the conformation of αE-catenin from a closed to an open state, allowing it to recruit vinculin to further reinforce cadherin adhesive forces (Figure 1). p120-catenin (encoded by *CTNND1*) binds to the “juxtamembrane domain” of the cadherin cytoplasmic tail to support cadherin stability, *cis* lateral clustering, and E-cadherin *trans* affinity (Yap et al., 1998; Thoreson et al., 2000; Ireton et al., 2002; Davis et al., 2003; Xiao et al., 2003; Davis and Reynolds, 2006; Kourtidis et al., 2013; Vu et al., 2021). Mature AJs form at apical regions of polarized epithelia, at the zonula adherens (ZA) (Nishimura and Takeichi, 2009). E-cadherin is considered a master regulator of the epithelial phenotype, due in part to its role in associating the ZA with a circumferential actomyosin ring that stabilizes the epithelial architecture (Miyoshi and Takai, 2008). Additionally, p120-catenin regulates the cytoskeleton via RhoGTPases (Anastasiadis, 2007), while the ZA interacts with microtubules either via β-catenin and dynein (Ligon et al., 2001) or via p120-catenin (Chen et al., 2003; Yanagisawa et al., 2004) and its interacting partner PLEKHA7 (Meng et al., 2008; Paschoud et al., 2014).

## Effects of deregulated cadherin-catenin signaling on major oncogenic pathways

### Contact inhibition and Hippo-YAP/TAZ signaling

In multicellular organisms, cell proliferation is tightly controlled by signals in the microenvironment and adjoining cells. Normal diploid cells in culture grow and divide in response to nutrients and growth factor signaling until they reach confluence. As cell contact increases in a density-dependent manner, cell proliferation slows down and is eventually halted in a process referred to as contact inhibition of proliferation (CIP) (Levine et al., 1965; Eagle and Levine, 1967; Hanahan and Weinberg, 2000; Motti et al., 2005; Hanahan and Weinberg, 2011). CIP is crucial for tissue morphogenesis and organ development. Loss of CIP allows cells to overgrow in epithelial monolayers, altering tissue architecture and leading to tumor formation (Levine et al., 1965). Hence, loss of CIP is one of the hallmarks of cancer and can deregulate signaling pathways that are normally suppressed by cell-cell interactions (Hanahan and Weinberg, 2000; Hanahan and Weinberg, 2011).

In an early study to identify molecular mechanisms underlying CIP, Whittenberger and Glaser observed that membrane isolates from cell monolayers were able to decrease proliferation of cells at sub-confluence (Whittenberger and Glaser, 1977). Since cadherins localize to the plasma membrane and mediate cell-cell interactions, they are prime candidates to initiate CIP. In agreement, re-expression of E-cadherin in cancer cells lacking endogenous expression suppressed cell proliferation via upregulation of cyclin-dependent kinase inhibitor p27 and reduction of cyclin E-cdk2 activity (St Croix et al., 1998; Motti et al., 2005). Addition of E-cadherin-neutralizing antibodies to these E-cadherin transfected cells repressed E-cadherin-mediated growth inhibition (St Croix et al., 1998). Moreover, re-expression of α-catenin in a lung adenocarcinoma cell line (PC9) lacking endogenous α-catenin rescued E-cadherin-mediated growth inhibition (Watabe et al., 1994). Another study reported that β-catenin binding to E-cadherin and E-cadherin engagement in subconfluent cells are sufficient to suppress growth in control or EGF treated cells (Perrais et al., 2007). Collectively these studies argue that E-cadherin-based adhesions contribute to CIP.

**FIGURE 2:**
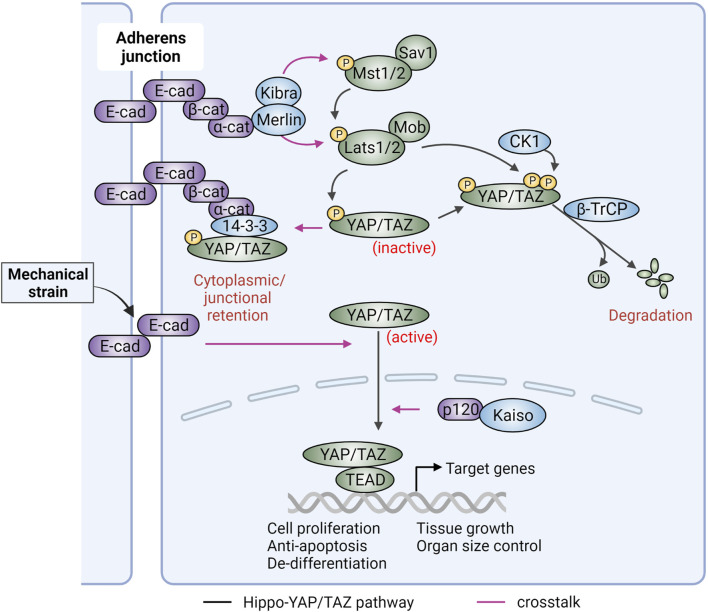
Hippo-YAP/TAZ signaling and its regulation by adherens junctions. Hippo-YAP/TAZ signaling includes the upstream Hippo components, which suppress activation of the downstream YAP/TAZ transcriptional coactivators. The core components of Hippo signaling include Mst1/2 and Lats1/2 kinases in complex with Sav1 and Mob scaffold proteins. Activated Lats1/2 promotes YAP/TAZ serine inhibitory phosphorylation, resulting in their cytoplasm/junctional retention (via 14-3-3) or β-TrCP mediated proteolytic degradation [if YAP/TAZ are further phosphorylated by casein kinase (CK1)]. Active, unphosphorylated YAP/TAZ can shuttle into the nucleus, where they cooperate with TEAD transcription factors to promote tumorigenic gene expression. This activation can be achieved by Hippo kinase inactivation, activation of nuclear p120-catenin (p120)/Kaiso signaling, or mechanical strain exerted at E-cadherin (E-cad) cell-cell junctions. In the absence of force, cadherin-mediated junctions can activate Hippo pathway kinases via β-catenin (β-cat) and α-catenin (α-cat) to inhibit YAP/TAZ-induced pro-tumorigenic signaling. Alpha-catenin can interact with YAP/TAZ and 14-3-3 preventing YAP/TAZ nuclear translocation. Merlin, which is linked to adherens junctions via α-cat, functions in a complex with the Kibra tumor suppressor to promote contact inhibition of proliferation by regulating Hippo-YAP/TAZ signaling. Figure created with BioRender.com.

As cell proliferation requires transcriptional regulation of cell cycle related genes, one immediate question regarding CIP is how cadherins and AJs at the plasma membrane transduce growth signals to the nuclear transcriptional machinery. Recent studies indicate a key role for the Hippo-YAP/TAZ signaling pathways. Nuclear accumulation of the transcriptional cofactors YAP and/or TAZ promotes cell cycle progression while Hippo signaling suppresses YAP/TAZ nuclear accumulation and inhibits cell growth (Zhao et al., 2008; Piccolo et al., 2014). In mammalian cells, the Hippo pathway includes the serine threonine protein kinases Mst1/2 (homologs of *Drosophila* Hippo) and Lats1/2 (homologs of *Drosophila* Warts) and their associated regulatory scaffold proteins Sav and Mob (Figure 2). Activation of Lats kinases by Mst results in inhibitory phosphorylation of YAP/TAZ (homologs of *Drosophila* Yorkie and WWTR1, respectively) (Meng et al., 2016; Fu et al., 2017). Once phosphorylated, YAP/TAZ undergo cytoplasmic retention via 14-3-3 interaction or proteasome-mediated degradation. When the Hippo kinases are suppressed, YAP/TAZ become active and can interact with transcription factors (TFs), such as TEAD proteins, following nuclear translocation, to transactivate a variety of pro-proliferative and anti-apoptosis genes. Consistent with a role for this pathway in CIP, YAP phosphorylation is increased with cell density (Zhao et al., 2007). Moreover, overexpression of YAP de-represses cell proliferation in high density culture and expression of a dominant negative YAP mutant rescues the CIP phenotype in cells with Hippo pathway deficiency (Zhao et al., 2007).

Given their roles in CIP, molecular alterations promoting activation of YAP/TAZ or inhibition of Hippo kinases would be expected in human cancers. While only a few somatic or germline mutations in the Hippo-YAP/TAZ pathway have been identified (Harvey et al., 2013; Huyghe et al., 2019), homozygous deletions and inactivating mutations of the Hippo kinase *LATS2* were reported in malignant mesothelioma (Murakami et al., 2011). Merlin (encoded by *NF2*), which activates Hippo kinases (Hamaratoglu et al., 2006; Zhang et al., 2010; Yin et al., 2013), is also frequently mutated in neurofibromatosis (Asthagiri et al., 2009) and malignant mesothelioma (Murakami et al., 2011). DNA amplification and upregulation of *YAP/TAZ* was seen in various cancers, such as squamous cancers and pancreatic cancer (Yu et al., 2015). In line with these findings, preclinical studies largely support the notion that deregulation of Hippo-YAP/TAZ signaling promotes cancer development [reviewed in (Harvey et al., 2013)]. Moreover, upregulation of a YAP/TAZ gene signature was found to be associated with worse clinical outcomes and chemotherapy resistance in cancer (Rozengurt et al., 2018; Nguyen and Yi, 2019).

A direct relationship between E-cadherin-mediated CIP and YAP/TAZ signaling was revealed using cells where E-cadherin engagement and CIP were triggered by E-cadherin-coated beads. Under these conditions, knockdown of Hippo core kinases or overexpression of YAP (wild-type and non-phosphorylatable active mutant) were both able to reverse E-cadherin mediated CIP (Kim et al., 2011). Conversely, expression of E-cadherin in cells lacking endogenous E-cadherin promoted cytoplasmic retention of YAP (Kim et al., 2011). Furthermore, α- and β-catenin, but not p120-catenin, were required for E-cadherin’s ability to retain YAP in the cytoplasm under conditions of high density (Kim et al., 2011). Mechanistically, an association between phosphorylated YAP and α-catenin via 14-3-3 in a tripartite complex was reported in epidermal keratinocytes (Schlegelmilch et al., 2011) (Figure 2). In this case, α-catenin functions as a negative upstream regulator of YAP. Upon calcium-mediated cell adhesion, YAP is associated with α-catenin at cellular junctions and knockdown of α-catenin leads to reduced phosphorylation and increased nuclear localization of YAP. Animal experiments with α-catenin gene targeting further argue that the tumor suppressing effects of α-catenin can be attributed to dysregulation of YAP activity (Silvis et al., 2011). This hypothesis is also supported by an inverse correlation between the levels of α-catenin and nuclear YAP in squamous cell carcinoma (Silvis et al., 2011). A role of α-catenin in suppressing YAP activity was also reported in endothelial cells, modulated by the actin processing protein EPS8. In cells with nascent junctions, YAP was active and able to shuttle into the nucleus as its binding to α-catenin was prevented by EPS8. Conversely, in mature junctions, YAP was phosphorylated by AKT downstream of VE-cadherin clustering and recruited to endothelial junctions via 14-3-3 and α-catenin (Giampietro et al., 2015). The data argue that α-catenin is a negative regulator of YAP signaling in both E-cadherin and VE-cadherin based cell-cell junctions.

Of note, α-catenin can bind directly to Merlin, a membrane-associated scaffold protein that functions as a tumor suppressor. This interaction is critical for AJ maturation (Gladden et al., 2010). Several studies have shown that Merlin in association with Kibra acts upstream of Hippo kinases (Hamaratoglu et al., 2006; Yin et al., 2013) (Figure 2). The biological impact of the connection between cadherins/catenins and Merlin was highlighted by a recent study in human mesothelioma cells showing that E-cadherin adhesions activate Merlin-Hippo signaling to resist ferroptosis, a form of cell death regulated by cellular metabolism and cellular iron (Wu et al., 2019). As several cancer-targeted agents induce ferroptosis (Lei et al., 2022), molecular alterations in cadherin/catenin and/or Merlin-YAP signaling could serve as biomarkers predicting cancer cell responsiveness to ferroptosis-inducing therapies.

As with α-catenin, β-catenin at junctions can induce YAP phosphorylation and cytoplasmic retention (Kim et al., 2011). However, the relationship between Hippo-YAP/TAZ signaling and β-catenin is more complex as β-catenin can shuttle between different subcellular pools (i.e., the plasma membrane, cytoplasm, and nucleus) in response to cellular and environmental cues (Henderson and Fagotto, 2002; Bienz, 2005; Krieghoff et al., 2006; Wu et al., 2008; Phelps et al., 2009). The most studied scenario is how WNT and Hippo-YAP/TAZ signaling regulate each other via β-catenin (see WNT section). Moreover, the impact of mechanical forces across E-cadherin junctions on β-catenin and YAP interaction has been examined in quiescent epithelial monolayers experiencing varying degrees of mechanical strain. In the absence of external stretching, YAP1 is localized to the cell cortex and cytoplasm and β-catenin is restricted at cell junctions. In response to mechanical strain that triggers E-cadherin engagement, both YAP1 and β-catenin translocate into the nucleus, with YAP1 driving cell cycle re-entry and β-catenin mediating cell transition into the S phase for DNA replication (Benham-Pyle et al., 2015) (Figure 2). Additionally, the association of nuclear p120-catenin with TF Kaiso, suppresses Kaiso’s transcriptional repressing activity resulting in the nuclear accumulation of YAP/TAZ through a mechanism that involves suppression of YAP/TAZ phosphorylation (Zhu et al., 2012) (Figure 2).

The effect of cadherin-catenin complexes on Hippo-YAP/TAZ signaling also extends to non-epithelial cells. Mesenchymal stem cells (MSCs) are pluripotent adult stromal cells that can differentiate into distinct cell lineages upon different mechanical cues and this process is known to be heavily regulated by YAP/TAZ. Interestingly, these cells can form “mechanical memory” by “remembering” the mechanical forces applied to them. Cells that have experienced stiff environment for some time (∼10 days) maintain nuclear accumulation of YAP/TAZ, even when replated onto more compliant substrates (Yang et al., 2014; Totaro et al., 2018). Using a hydrogel-based system capable of mimicking N-cadherin based cell-cell adhesions (using HAVDI peptide derived from the EC1 repeat of N-cadherin) and integrin-mediated cell-extracellular matrix (ECM) interactions (using RGD peptides), cell-ECM adhesions were found to mediate the perception and retention of mechanical memory via nuclear YAP. This feature was reversed by N-cadherin ligation and junctional β-catenin, which promoted re-localization of nuclear YAP to the cytoplasm (Zhang et al., 2021).

In summary, available data suggest that CIP is at least in part mediated by CCC-induced downregulation of YAP/TAZ signaling. E-cadherin can directly restrain YAP1 nuclear translocation via its extracellular engagement (Benham-Pyle et al., 2015), and also promote Hippo kinase activity to repress YAP/TAZ mediated transcription through its cytoplasmic domain associated catenins (Kim et al., 2011). Further, CCCs can function as a nexus to transduce mechanical cues to Hippo-YAP/TAZ signaling. As the majority of these studies utilized simple cell line models, studies in model organisms, patient-derived organoids [reviewed in (Kim et al., 2020)], or tumor-on-a-chip models [reviewed in (Sontheimer-Phelps et al., 2019; Liu et al., 2021)] could provide further insight into the interplay between CCC and Hippo-YAP/TAZ signaling and its role in cancer.

### Receptor tyrosine kinase signaling pathways

**FIGURE 3:**
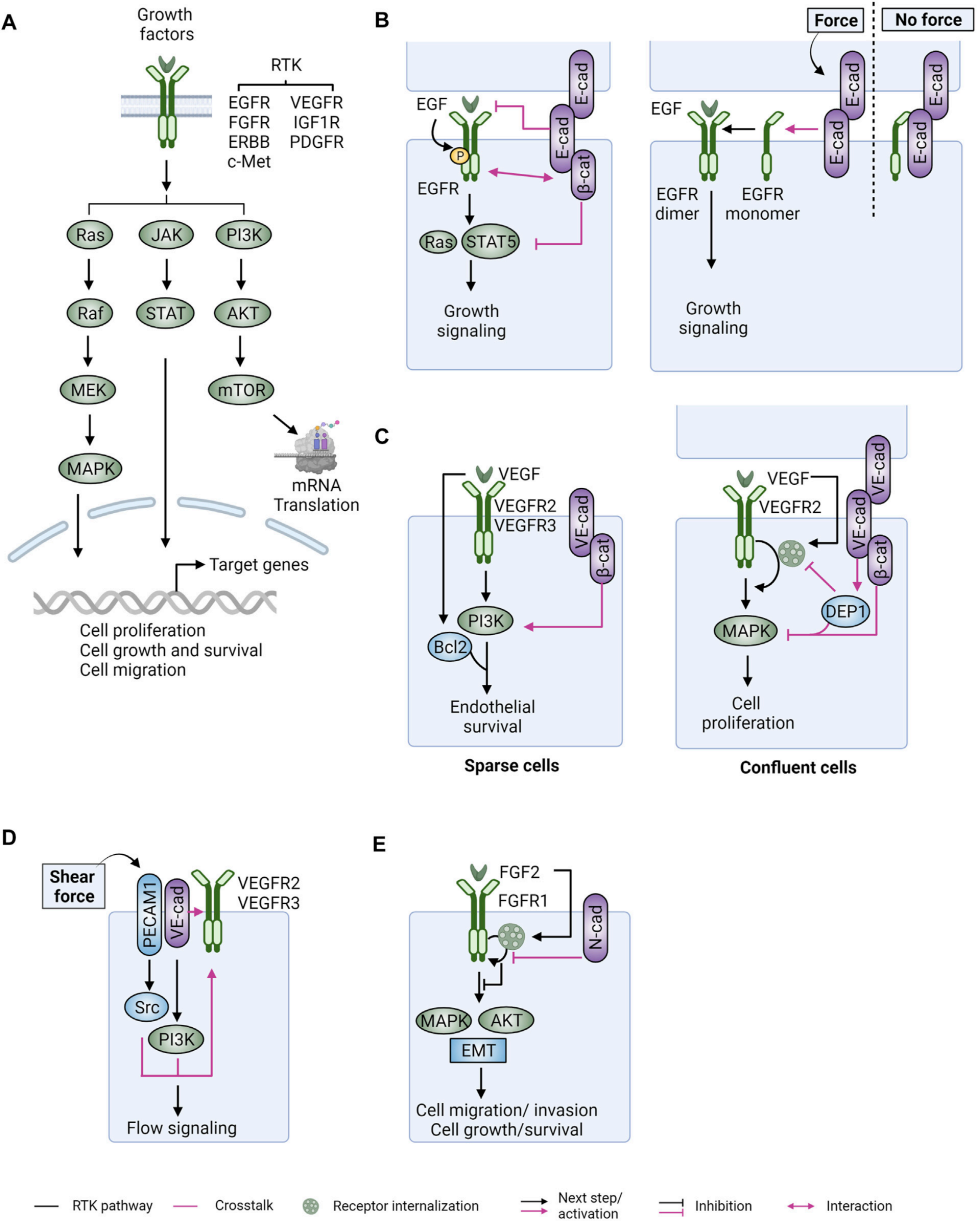
Crosstalk between receptor tyrosine kinases and cadherin-catenin complexes. **(A)** RTK binding to their cognate ligands (i.e., growth factors) and subsequent receptor dimerization activates three major downstream pathways (RAS-RAF-MEK-MAPK, JAK-STAT, and PI3K-AKT-mTOR) that regulate gene expression and various cellular processes. **(B)** (Left) In epithelial cells, E-cadherin (E-cad) can associate with EGFR through its lateral ectodomains and/or intracellular β-catenin (β-cat). E-cadherin ligation and its interaction with β-catenin are required for inhibition of EGF/EGFR-mediated cell growth via STAT5 and RAS. In general, E-cadherin junctions suppress EGFR activation and the conveyance of signal transduction to downstream effectors, while EGFR activation can promote the disassembly of E-cadherin junctions. (Right) EGFR is sequestered by E-cadherin in the absence of force, whereas it is released to set off growth signaling when external force is applied to E-cadherin junctions. **(C)** (Left) In endothelial cells with sparse cell contacts (sparse), VEGF-A ligand activated VEGFR2 associates with vascular adherens junctions via direct interaction with VE-cadherin (VE-cad) or through a linkage between PI3K and β-cat, resulting in increased endothelial proliferation (via VEGF-A induced Bcl2 upregulation), mechanotransduction, and angiogenesis. (Right) In confluent cells, junctional phosphatase DEP1 is recruited to VE-cad/β-cat junctions to inhibit VEGFR signaling by suppressing its receptor internalization and transduction to MAPK. **(D)** VEGFR2/3 can be activated upon shear stress in the absence of VEGF ligands. In this scenario, a tripartite complex of PECAM1, VE-cad, and VEGFR is formed. Activation of Src and PI3K kinases, downstream of PECAM1 and VE-cad, respectively, potentiates VEGFR signaling, collaboratively contributing to flow signaling. **(E)** In tumor cells, interaction of N-cadherin (N-cad) and fibroblast growth factor receptors (FGFRs) upon ligand stimulation sustains RTK signaling to promote cell motility and invasion. Figure created with BioRender.com.

RTKs and their downstream signaling pathways are crucial for cell proliferation, differentiation, and survival during development (Schlessinger, 2014) (Figure 3A). Activation of RTK signaling is common in cancer, and often attributed to amplifications, mutations, rearrangements, and overexpression of pathway components. For example, activating mutations and amplifications of epithelial growth factor receptors (*EGFR*s) are frequently identified in glioma (Wong et al., 1992; Cancer Genome Atlas Research Network, 2008) and lung adenocarcinoma (Cancer Genome Atlas Research Network, 2014b). *ERBB2* (also known as HER2) is a commonly amplified and overexpressed proto-oncogene in a subset of breast cancer (Slamon et al., 1987; Slamon et al., 1989; Cancer Genome Atlas Network, 2012). Aberrations of fibroblast growth factor receptors (*FGFRs*), c-Met, and insulin-like growth factor 1 receptor (*IGF1R*) are frequently found in urothelial carcinoma (Cancer Genome Atlas Research Network, 2014a; Helsten et al., 2016), hepatocellular carcinoma (HCC) (Park et al., 1999; Cancer Genome Atlas Research Network, 2017) and breast cancer (Cancer Genome Atlas Network, 2012; Farabaugh et al., 2015), respectively. In addition to these growth factor signaling pathways that are activated in cancer cells, vascular endothelial growth factor receptor (VEGFR) signaling is frequently activated in endothelial cells by vascular endothelial growth factor (VEGF) released from tumor cells and stroma to promote neo-angiogenesis to facilitate tumor growth (Apte et al., 2019).

Support for crosstalk between CCCs and RTKs was initially provided by observations that catenins are phosphorylated in response to growth factors (Hoschuetzky et al., 1994; Shibamoto et al., 1994; Hazan and Norton, 1998). Different modes of physical interaction between CCCs and EGFR have been reported (Figure 3B). The EGFR can associate with CCCs through interaction with β-catenin (Hoschuetzky et al., 1994; Perrais et al., 2007). E-cadherin can also interact with EGFR via ECs without binding to β-catenin (Qian et al., 2004). The interaction between these two signaling hubs allows a reciprocal regulation, by which EGFR promotes cell junction disassembly in part due to increased endocytosis of CCCs (Hazan and Norton, 1998; Fujita et al., 2002), whereas E-cadherin adhesions modulate activation and cellular signaling of EGFR in a cell-cell contact and/or cell density dependent manner (Pece and Gutkind, 2000; Qian et al., 2004; Perrais et al., 2007). In confluent epithelial cells, E-cadherin junctions are generally thought to suppress EGFR activation and downstream RAS signaling by sequestering EGFR away from apical cell domains, thus suppressing EGF binding to EGFR (Takahashi and Suzuki, 1996; Qian et al., 2004) (Figure 3B, left). Consistent with its ability to promote E-cadherin stability, p120-catenin through its interaction with E-cadherin suppresses overall tyrosine phosphorylation levels in epithelial cells and inhibits RAS signaling (Soto et al., 2008). Moreover E-cadherin binding to β-catenin, despite not affecting autophosphorylation of EGFR, suppresses EGFR-mediated STAT5 activation (Perrais et al., 2007) (Figure 3B, left). In response to mechanical stress at E-cadherin junctions, EGFR growth signaling becomes active followed by its dissociation with E-cadherin (Sullivan et al., 2022) (Figure 3B, right). EGFR further functions as a positive regulator for force-mediated E-cadherin signaling that increases cell stiffness through the activation of PI3K and Abl kinases (Sehgal et al., 2018). Interestingly, the basolateral localization and apical exclusion of EGFR in polarized epithelial cells may exclude EGF, but promote signaling by alternate ligands, like amphiregulin or epiregulin, which localize exclusively to the basolateral domain (Damstrup et al., 1999; Singh et al., 2013). Finally, E-cadherin complexes also associate with and are reciprocally regulated by other RTKs such as c-Met, HER2, and IGF1R (Gamallo et al., 1993; Moll et al., 1993; Shibamoto et al., 1994; Hiscox and Jiang, 1999; Kamei et al., 1999; Fujita et al., 2002; Cozzolino et al., 2003; Yanagisawa et al., 2004; Nagle et al., 2018). Collectively, these studies indicate the presence of intricate crosstalk between CCCs and RTK signaling that is likely critical for cell growth and tissue homeostasis (Kim et al., 2009).

This reciprocal relationship is clearly evident in the *Drosophila* gut where healthy enterocytes suppress stem cell division through the repression of EGF secretion by E-cadherin. Conversely, dying cells activate EGFR signaling by losing E-cadherin to increase cell division. This coordinated feedback regulation ensures that the total number of cells in the organ remain constant and thus prevents hyperplasia or atrophy (Liang et al., 2017; Ngo et al., 2020). The interaction of CCCs with Merlin provides an additional mechanism of EGFR pathway regulation. Upon cell-cell contact, the adaptor protein Na+/H+ exchanger regulatory factor 1 (NHERF1) links EGFR to Merlin, which sequesters the receptor at the cortical membrane to prevent its internalization and activation (Curto et al., 2007; Cole et al., 2008). At the plasma membrane-cytoskeleton interface, lateral mobility and recycling of EGFR within the membrane is restrained by a coordinated effort involving Merlin, cortical actomyosin cytoskeleton, and the composition of membrane lipids (Chiasson-MacKenzie et al., 2015; Chiasson-MacKenzie et al., 2018). Perturbation of this inhibitory effect of Merlin on EGFR signaling promotes tumorigenesis in the mouse liver (Benhamouche et al., 2010).

Recently, a new E-cadherin variant was found to be overexpressed in glioblastoma and to affect EGFR signaling in an unprecedented manner. This variant is encoded by E-cadherin circular RNA (circRNA) and exhibits distinct functions from the common E-cadherin encoded by linear RNA (Gao et al., 2021). Circular RNAs (circRNAs) encompass one or multiple exons, are more stable than linear RNAs, and are often considered as miRNA sponges that shield mRNAs from miRNA-dependent degradation (Kristensen et al., 2019). While full-length E-cadherin is low or absent in brain tumor cells, expression of variant E-cadherin from circRNAs was high, was secreted by glioma stem cells, and acted as an autocrine ligand to amplify EGFR signaling independent of EGF, resulting in sustained glioblastoma stemness (Gao et al., 2021).

Other cadherins can also modulate the function of growth factor receptors (GFRs). In endothelial cells, VEGF and its cognate receptors (VEGFRs) control cell survival, proliferation, and angiogenesis [reviewed in (Koch and Claesson-Welsh, 2012)]. Upon VEGF binding, VEGFR-2 (also known as FLK1, encoded by *KDR*) can form physical interactions with VE-cadherin through β-catenin to modulate downstream signaling events in a cell density-dependent manner (Carmeliet et al., 1999; Grazia Lampugnani et al., 2003). In cells with reduced cell contacts, this complex (consisting of VEGFR-2, VE-cadherin, and β-catenin) recruits PI3K to activate Akt and involves Bcl-2 to drive endothelial survival and proliferation (Carmeliet et al., 1999) (Figure 3C, left). In confluent cells, junctional phosphatase CD148/DEP-1 is recruited to this complex upon VE-cadherin engagement to suppress VEGFR-2 phosphorylation and internalization, thus inhibiting endothelial proliferation (Grazia Lampugnani et al., 2003; Lampugnani et al., 2006) (Figure 3C, right).

On the other hand, VEGFRs can be activated by shear flow in a ligand-independent manner (Jin et al., 2003). Shear force in the vasculature is generated by the blood flowing through the vessels, imparting physical forces on endothelial cells which comprise vessel walls. Under this condition, the complexes of junctional VE-cadherin with VEGFRs (VEGFR-2 and VEGFR-3) function as a crucial node that transmits flow signals, and converts them into biochemical responses (Shay-Salit et al., 2002; Tzima et al., 2005; Coon et al., 2015; Conway et al., 2017). The VE-cadherin-VEGFR2 associated complex can also be coupled with another adhesion receptor PECAM-1 to transmit shear stress signals, leading to the activation of Src kinases and the PI3K pathway (Shay-Salit et al., 2002; Tzima et al., 2005; Conway et al., 2013) (Figure 3D). Interestingly, the mechanotransduction mediated by local VE-cadherin adhesions and the downstream intercellular junctional remodeling can be propagated to junctions distal to where the force is applied (Barry et al., 2015).

VEGF and its cognate receptors are crucial for neo-angiogenesis. Interestingly, in ovarian cancer, VE-cadherin on endothelial cells can form a direct heterophilic interaction with a form of soluble E-cadherin (sE-cadherin) localized on the surface of exosomes. sE-cadherin is the cleaved-off ectodomain of E-cadherin and is highly expressed in malignant ascites as well as tumor cells with high metastasis capabilities. This unique interaction between endothelial cells and exosomes leads to angiogenesis via VEGF-independent activation of Wnt/β-catenin and NF-κB signaling (Tang et al., 2018). Due to the close crosstalk between VE-cadherin and VEGFR-2, an interesting question to ask is whether sE-cadherin/VE-cadherin interactions promote angiogenesis by affecting VEGFR-2 signaling.

In tumor cells, N-cadherin is frequently upregulated, enhancing migratory and invasive cellular behaviors (reviewed in (Derycke and Bracke, 2004). Inspired by the synergistic activity of N-cadherin and FGFR in neurite outgrowth (Williams et al., 1994), the relationship between the two in cell motility and invasion was explored. Molecularly, FGFR signaling is sustained in the presence of N-cadherin as FGF-2 stimulation brings N-cadherin and FGFR1 together to inhibit ligand-mediated receptor internalization, resulting in increased stability of FGFR1 and cell motility/invasiveness (Suyama et al., 2002; Hulit et al., 2007) (Figure 3E). In line with these findings, expression of N-cadherin in mouse cancer models supports the notion that N-cadherin and FGFR synergistically potentiate downstream MAPK and AKT signaling with EMT to promote cancer metastasis (Shintani et al., 2006; Hulit et al., 2007; Su et al., 2012; Qian et al., 2014). Furthermore, β-catenin association with NHERF2 induced the interaction of N-cadherin with platelet derived growth factor receptor beta (PDGFR-β) to promote cell motility (Theisen et al., 2007).

Cadherin-associated catenins have also been shown to play a vital role in growth factor signaling which involves signal transduction from the upstream GFRs to the downstream Ras-MAPK pathway. In mouse skin with α-catenin ablation, a sustained upregulation of Ras-MAPK signaling was found in keratinocytes. Detailed analyses showed that there is no upregulation of RTK levels and/or activity in these knockout cells despite an increased sensitivity to insulin and IGF-1 and activation of Ras-MAPK. Upon insulin stimulation of α-catenin null cells, a physical association between the E-cadherin/β-catenin complex and phosphorylated insulin receptor substrate 1 (IRS-1) is formed. This potentiates the propagation of IGF1R signaling to Ras-MAPK, promoting epidermal hyperplasia (Vasioukhin et al., 2001). In early stage hepatocellular carcinoma (HCC), the AJ-associated β-catenin is reported to increase EGFR stability, promoting tumor growth (Kim et al., 2019). p120-catenin, on the other hand, inhibits Ras when binding to E-cadherin but promotes Ras-MAPK activation and tumor cell growth when associated with mesenchymal cadherins upon E-cadherin loss (Soto et al., 2008). p120-catenin also plays key roles in EGF- and HGF-mediated cell migration/invasion and scattering via its ability to modulate the activities of Rac and Rho small GTPases (Cozzolino et al., 2003; Yanagisawa et al., 2008). Moreover, a study in *Drosophila* showed that nuclear p120-catenin can induce expression of the EGF maturation factor rhomboid (Liang et al., 2017), whose mammalian paralog RHBDD1 promotes colorectal cancer tumorigenesis via EGFR-RAS-MAPK signaling (Song et al., 2015).

From an evolutionary standpoint, it is notable that the transition to multicellularity is marked by the simultaneous addition of adhesion proteins, including cadherins, and RTKs in choanoflagellates (King et al., 2003; Abedin and King, 2008). The reciprocal crosstalk between these two receptor classes fine tunes both adhesion and growth mediated responses through mechanisms that can be dependent or independent of cell contacts and/or GFR ligands and is commonly deregulated in cancer. RTK alterations are one of the most prominent driving events in human cancer (Sanchez-Vega et al., 2018) and prime targets for cancer therapy [reviewed in (Yamaoka et al., 2018; Pottier et al., 2020)]. Unfortunately, crosstalk between different RTKs often mitigate tumor cell response to a given RTK inhibitor. As CCCs collectively regulate the function of multiple RTKs, a better understanding of their crosstalk could provide novel mechanistic and therapeutic insights for the treatment of cancer.

### Oncogenic Wnt/β-catenin signaling

The Wnt signaling pathway is essential for tissue homeostasis, embryonic development, and stem cell regeneration and maintenance, and commonly categorized into canonical and non-canonical forms based on β-catenin dependence [Grumolato et al., 2010; Zhan et al., 2017)]. Here, we focus on β-catenin dependent, canonical Wnt signaling (as known as Wnt/β-catenin signaling), which is one of the key cancer driver pathways (Sanchez-Vega et al., 2018). For readers with a particular interest in non-canonical Wnt, several comprehensive reviews are available (Kohn and Moon, 2005; Wang, 2009; Akoumianakis et al., 2022). Notably, non-canonical Wnt signaling is suppressed by the transcriptional repressor Kaiso, and this suppression is relieved by association of Kaiso with p120 catenin (Kim et al., 2004).

**FIGURE 4:**
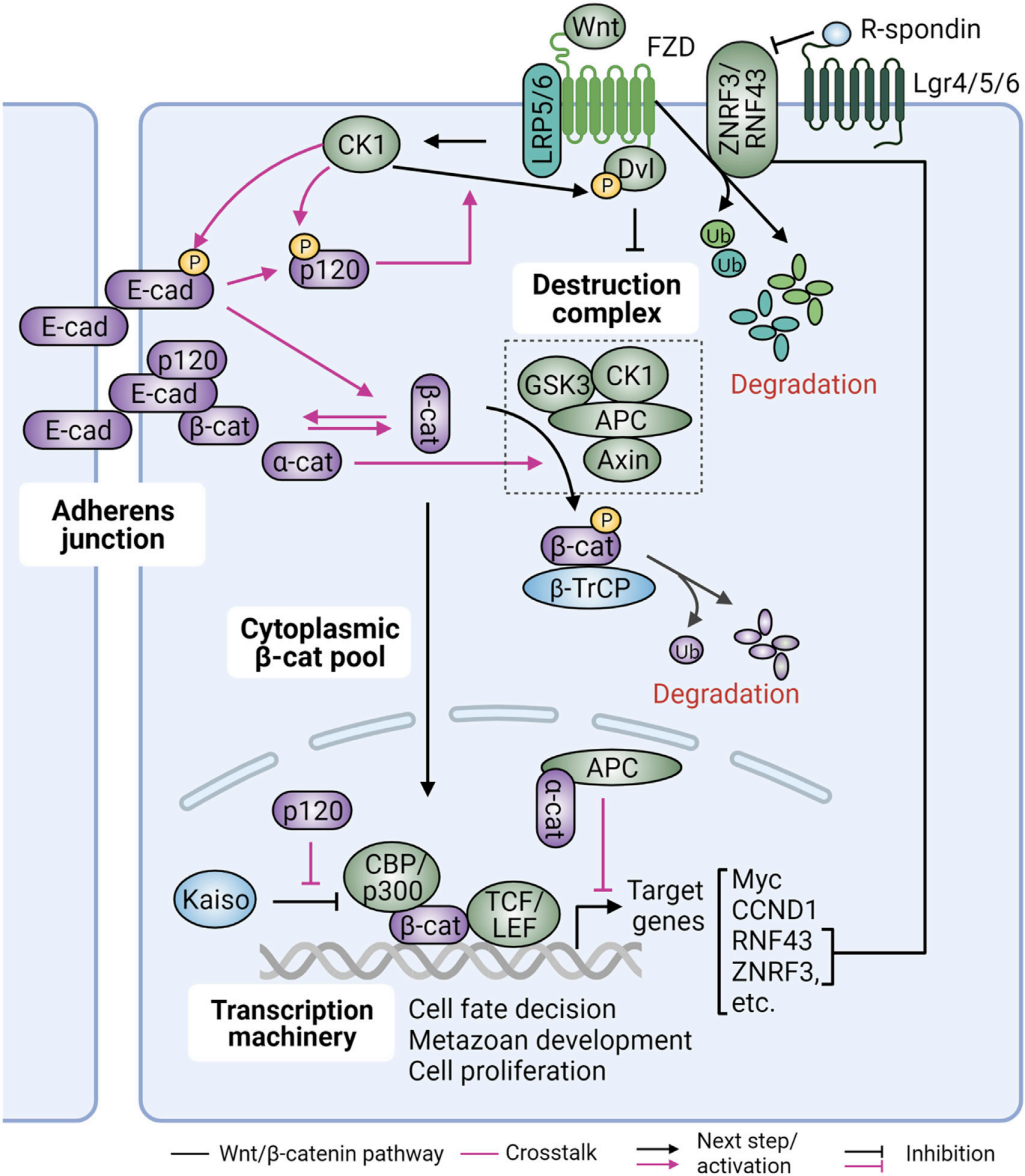
Interrelationship between Wnt/β-catenin signaling and cadherins/catenins. Canonical Wnt signaling is a crucial regulator of cell fate determination, cell proliferation and embryonic development. It is kept inert when cytoplasmic β-catenin (β-cat) levels are low by the action of the multiprotein Destruction Complex and β-TrCP-mediated proteasomal degradation. Wnt ligand binding to Frizzled (FZD) receptors and low-density lipoprotein receptor-related protein 5/6 (LRP5/6) co-receptors results in dishevelled (Dvl) recruitment and activation by casein kinase (CK1), leading to β-cat release from the Destruction Complex. The freed cytoplasmic β-cat is then translocated into the nucleus where it interacts with transcription (co)factors TCF/LEF and CBP/p300 to drive gene expression. Wnt signaling is also negatively regulated by RNF43 and ZNRF3 ubiquitin E3 ligases, which degrade FZD and LRP receptors. Conversely, R-spondin and Dvl positively regulate Wnt signaling by binding to Lgr4/5/6 receptors to remove RNF43/ZNRF3 from the plasma membrane, or by inhibiting the β-cat Destruction Complex, respectively. Upon Wnt stimulation, E-cadherin (E-cad) is phosphorylated by CK1 resulting in dissociation of β-cat, which can then participate in Wnt signaling or be degraded. This process can be induced by cytoplasmic α-catenin (α-cat) via increased β-cat binding to adenomatous polyposis coli (APC). Additionally, nuclear α-cat can recruit APC to the β-cat-TCF/LEF complex to suppress expression of Wnt target genes. P120-catenin (p120), on the other hand, is also phosphorylated by CK1, leading to its dissociation from E-cadherin and increased formation of the Wnt signalosome via interaction with LRP5/6. Finally, p120 in the nucleus can de-repress Kaiso’s action on β-cat/TCF-TEF activity. Figure created with BioRender.com.

In canonical Wnt signaling, β-catenin is a key downstream effector to promote transcription of Wnt target genes (Figure 4). In the absence of Wnt ligands (Wnt OFF), the levels of cytosolic β-catenin are kept low by the Destruction Complex, which contains the tumor suppressors Axin and adenomatous polyposis coli (APC), and the serine/threonine kinases glycogen synthase kinase 3β (GSK-3β) and casein kinase 1 (CK1) (Zhan et al., 2017). In this complex APC serves as a platform, allowing β-catenin to interact with other proteins. GSK3β and CK1 phosphorylate β-catenin to prime its recognition by the E3-ubiquitin ligase β-TrCP, marking β-catenin for degradation by the proteasome. In Wnt/β-catenin signaling (Wnt ON), trafficking and secretion of Wnt ligands are enabled by the Porcupine acyltransferase and Wntless membrane protein (Takebe et al., 2015). Secreted Wnt ligands then bind to Frizzled (FZD) family receptors and associated low-density lipoprotein receptor-related protein 5/6 (LRP5/6) co-receptors on neighboring cells to initiate Wnt signaling. Ligand-bound FZD receptors then recruit and activate dishevelled (Dvl) at the plasma membrane. Activated Dvl, in turn, recruits and inhibits the β-catenin Destruction Complex, leading to accumulation of β-catenin in the cytoplasm. Stabilized β-catenin translocates to the nucleus where it interacts with the T cell factor (TCF)/lymphocyte enhance factor (LEF) family of TFs to drive gene expression. The E3 ubiquitin ligases zinc and ring finger 3 (ZNRF3) and its homologue RNF43, which are Wnt target genes, in turn form a negative feedback circuit to target FZD receptors for lysosomal degradation (de Lau et al., 2011; Koo et al., 2012). R-spondin ligands, on the other hand, form a complex with the G-protein-coupled receptors (GPCRs) Lgr4/5/6 to maintain Wnt signaling via inhibition of ZNRF3/RNF43 (Hao et al., 2012; de Lau et al., 2014). In cancer, alterations of these molecules can lead to activation of the Wnt pathway, promoting cancer initiation and progression.

The primary mechanism leading to hyper-activation of Wnt signaling in cancer is loss of function (LoF) in *APC*, an event that is commonly observed in hereditary (familial adenomatous polyposis) and sporadic colorectal carcinomas (CRC) (Nishisho et al., 1991; Cottrell et al., 1992; Powell et al., 1992; Rowan et al., 2000). Dysregulation of APC negates its normal function to restrict β-catenin levels through the Destruction Complex (Rubinfeld et al., 1993; Morin et al., 1997). Activating mutations of *CTNNB1* (β-catenin) are also found in CRC and are mutually exclusive with *APC* alterations (Morin et al., 1997; Sparks et al., 1998). In endometrial carcinomas, mutations of *CTNNB1* (β-catenin) are the predominant mechanism that triggers Wnt/β-catenin signaling (Fukuchi et al., 1998; Cancer Genome Atlas Research Network et al., 2013). These mutational events often occur in the NH2-terminal regulatory domain (exon 3, in particular) of β-catenin to prevent its phosphorylation and subsequent ubiquitination and degradation (Gao et al., 2018). Intriguingly, accumulation of β-catenin and thus activation of Wnt signaling are also implicated in triple-negative breast cancer (TNBC) despite no evidence of *CTNNB1* mutations (Khramtsov et al., 2010; Geyer et al., 2011). Moreover, the R-spondin-ZNRF3/RNF43 module is often deregulated to activate Wnt signaling in cancer. R-spondin gene fusions can drive colon tumor formation and progression (Seshagiri et al., 2012; Han et al., 2017), whereas inactivating mutations of RNF43 confer sensitivity to porcupine inhibitors in Wnt-dependent CRC and endometrial tumor organoids (Giannakis et al., 2014; van de Wetering et al., 2015).

As β-catenin is an essential component of both AJs and canonical Wnt signaling, crosstalk between the two has long been suspected. Initial studies in *Xenopus* and *Drosophila* showed that cadherin overexpression can antagonize Wnt signaling by sequestering β-catenin to the plasma membrane (Heasman et al., 1994; Sanson et al., 1996) (Figure 4). Similar observations were also reported in cell culture systems overexpressing cadherins (Sadot et al., 1998; Gottardi et al., 2001; Stockinger et al., 2001). The competition between junctional and nuclear β-catenin can be explained by the observation that the interaction domains of β-catenin for cadherin, APC, and TCF overlap and thus interactions are mutually exclusive. Functionally, this implied that loss of E-cadherin during cancer progression would be sufficient to promote canonical Wnt signaling. However, the lack of upregulation of β-catenin signaling upon cadherin loss in several models argues that β-catenin released from cadherin complexes is rapidly degraded by the Destruction Complex, and additional events including Wnt activation and/or reduced degradation are required for the induction of β-catenin signaling (Mendonsa et al., 2018). Under these conditions, loss of E-cadherin could potentiate canonical Wnt signaling and contribute to cancer progression.

Recently, Wnt signaling was shown to interconnect with Hippo-YAP/TAZ signaling via β-catenin. Briefly, nuclear β-catenin forms a complex with active YAP to potentiate the transcriptional programs of both WNT/β-catenin and YAP pathways (Heallen et al., 2011; Rosenbluh et al., 2012). Moreover, cytoplasmic YAP/TAZ are recruited to the Destruction Complex by β-catenin, ensuring the recruitment of the β-TrCP E3 ubiquitin ligase for protein degradation (Azzolin et al., 2012; Azzolin et al., 2014). Consequently, both β-catenin and TAZ are maintained at low levels, preventing overactivation of WNT and YAP/TAZ pathways (Azzolin et al., 2012; Azzolin et al., 2014). These findings highlight multiple mechanisms that link Hippo-YAP/TAZ and Wnt/β-catenin pathways and suggest potential crosstalk with cadherin catenin complexes.

Finally, α- and p120-catenins also influence transcription of Wnt target genes (Figure 4). Cytoplasmic α-catenin physically associates with APC to promote ubiquitination and subsequent degradation of β-catenin. Upon Wnt3A stimulation, nuclear α-catenin recruits APC to canonical Wnt response elements to regulate β-catenin turnover (Choi et al., 2013). Independent of the destruction complex, nuclear α-catenin can also block the interaction of the β-catenin/TCF transcriptional complex with DNA, thus suppressing TCF-dependent transcription (Giannini et al., 2000). Cadherin-associated p120-catenin is phosphorylated by CK1ε in response to Wnt3a, which promotes recruitment of CK1 and Dvl2 to the Wnt co-receptors LRP5/6 (Casagolda et al., 2010). By promoting the formation of the Wnt signalosome, p120-catenin promotes β-catenin stability and transcriptional activity. Another mechanism by which p120-catenin promotes β-catenin signaling is by de-repressing transcription of Wnt target genes by promoting the dissociation of the transcriptional repressor Kaiso from the promoters of canonical Wnt target genes (Park et al., 2005).

In summary, in the absence of activating events (Wnt ligand, APC mutations, etc.) loss of E-cadherin alone is insufficient to promote Wnt/β-catenin signaling. However, in the presence of activating events, cadherins and catenins are able to modulate canonical Wnt signaling by acting at different subcellular locations and with various mechanisms of action to regulate signalosome formation, β-catenin degradation, or transcriptional activation.

### Notch and Hedgehog developmental and stemness pathways

**FIGURE 5:**
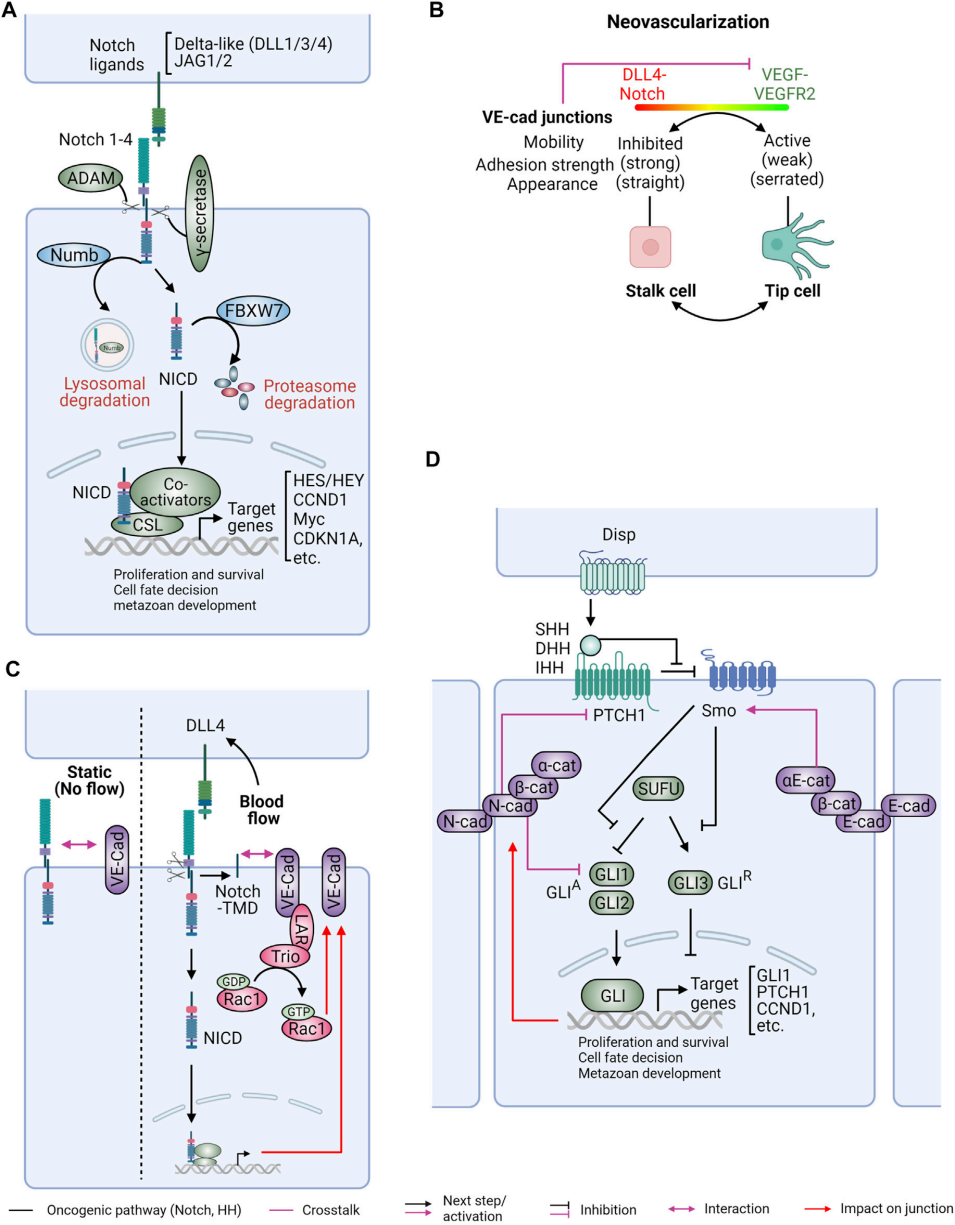
Interplay between Notch and Hedgehog signaling with cadherin/catenin complexes. Notch and hedgehog (HH) are key pathways for tissue homeostasis, embryonal development, and cancer stemness. **(A)** Notch signaling commences when transmembrane Notch ligands bind to the corresponding receptors on neighboring cells. This interaction results in proteolytic cleavage of Notch receptors by ADAMs and γ-secretase enzymes, releasing the Notch intracellular domain (NICD). The NICD translocates into the nucleus, where it functions as a transcriptional regulator to promote gene expression. Notch signaling can be negatively regulated by the E3 ubiquitin ligase specificity factor FBXW7 and the endocytosis regulator Numb. **(B)** During neovascularization, Dll4/Notch and vascular endothelial growth factor (VEGF)/VEGF receptor 2 (VGFR2) signaling cooperatively determine the stability and dynamics of junctional VE-cadherin (VE-cad) to drive the rearrangement of tip cells and stalk cells during angiogenic sprouts. Endothelial cells become stalk cells when they encounter high level of Notch signaling with low VEGFR2 activity. These cells exhibit reduced VE-cadherin junctions with stronger adhesions and straight appearance. In contrast, low Notch activity with high VEGFR2 signaling promotes the transformation of endothelial cells to tip cells. As a feedback mechanism, VE-cad association with VEGFR2 inhibits VEGFR2 signaling. **(C)** The interplay between VE-cad and Notch signaling can be modulated by mechanical signals. (Left) Under no flow static condition, VE-cad can associate with full-length Notch1. (Right) Shear stress, on the other hand, has been shown to induce both conventional transcription-dependent and unconventional transcription-independent Notch signaling with induced Dll4 expression. Increased expression of Notch target genes upon hemodynamic forces is critical for the maintenance of VE-cad junctions. Additionally, Notch cleavage can lead to release of its transmembrane domain (TMD), which forms a direct association with VE-cad to promote endothelial barrier function via the LAR phosphatase/Trio guanine-nucleotide exchange factor/Rac1 signaling axis. **(D)** In the absence of HH ligands, the activity of Smo is inhibited by the Patched 1 (PTCH1) receptor. Once HH ligands are released by the transporter Dispatched (Disp) at neighboring cells and binding to PTCH1, Smo becomes active and then inhibits Suppressor of Fused (SUFU), resulting in nuclear translocation of glioma-associated oncogene homolog (Gli) proteins. Gli proteins regulate expression of HH target genes with Gli1 and Gli2 being transcriptional activators (GLI^A^) and Gli3 acting as a transcriptional repressor (GLI^R^). Cadherin (E-cadherin, N-cadherin) adhesions restrict activation of HH signaling, in part through α-catenin (α-cat), although the underlying molecular mechanisms have not been fully elucidated. Activated HH signaling, in turn, promotes assembly of N-cad cell junctions. Figure created with BioRender.com.

Similar to Wnt signaling, Notch and HH play crucial roles in tissue development and homeostasis (Takebe et al., 2015). Extensive crosstalk between these three pathways collectively governs self-renewal and cell-fate decisions (Takebe et al., 2011; Takebe et al., 2015). The Notch signaling pathway begins with the interaction between transmembrane-bound Notch ligands and receptors on neighboring cells (Figure 5A). Five Notch ligands, Delta-like ligand 1 (DLL1), DLL3, DLL4, Jagged 1 (Jag1), and Jag2, and four Notch receptors (Notch1-4), are involved in this pathway. Different ligands and receptors are expressed in different tissues and tumor types [reviewed in (Takebe et al., 2011; Takebe et al., 2015)]. Upon ligand-receptor association, Notch receptors undergo a two-step proteolytic cleavage by the a-disintegrin and metalloproteinase (ADAM) enzymes (ADAM10 or ADAM17) and γ-secretase, releasing the Notch intracellular domain (NICD) (Gordon et al., 2007). Released NICD then translocates to the nucleus to interact with the DNA-binding transcription regulator RBPJ and transcriptional coactivators to activate or repress gene expression. The best-known Notch target genes include members of the Hes/Hey family of basic helix-loop-helix (βHLH) TFs, *CCND1* (encodes cyclin D1), *CDKN1A* (encodes p21) and Myc (Bailey and Posakony, 1995; Jarriault et al., 1998; Rangarajan et al., 2001; Ronchini and Capobianco, 2001; Iso et al., 2003; Klinakis et al., 2006; Weng et al., 2006). This signaling can be terminated by degradation of NICD by the E3 ubiquitin ligase F-box and WD repeat domain containing 7 (FBXW7) (Tsunematsu et al., 2004).

The first evidence linking Notch signaling to cancer was a chromosomal translocation targeting *Notch1* in T-cell acute lymphoblastic leukemia (T-ALL) (Ellisen et al., 1991). Later studies showed that activating mutations of *Notch 1* and inactivating mutations of the tumor suppressor gene (TSG) *FBXW7* were common in T-ALL (Weng et al., 2004; Malyukova et al., 2007; Maser et al., 2007; Thompson et al., 2007). These molecular alterations lead to constitutive activation of Notch signaling and drive neoplastic transformation. A pro-tumorigenic role for Notch signaling has also been reported in other hematological malignancies and solid tumors (Ranganathan et al., 2011; Misiorek et al., 2021). Activation of Notch signaling in these diseases can be also attributed to overexpression of Notch receptors and ligands and inactivating mutations of the negative regulators *FBXW7* and *Numb*. However, the role of Notch signaling in cancer is nuanced by observations that in some contexts it can act as a tumor suppressor. For example, Notch signaling promotes cell differentiation and inhibits proliferation in skin keratinocytes (Lowell et al., 2000; Rangarajan et al., 2001; Nicolas et al., 2003). A similar inhibitory effect on cell growth was also reported in small cell lung cancer (SCLC) (Sriuranpong et al., 2001). Two genome-wide association studies (GWAS) delineating the molecular mechanisms underlying head and neck squamous cell carcinoma (HNSCC) have identified inactivating mutations in *Notch1*, suggesting a tumor-suppressing function of Notch in this disease (Agrawal et al., 2011; Stransky et al., 2011). In contrast, activation of Notch target genes HES1/HEY1 has also been reported in HNSCC, suggesting a tumor promoting function (Sun et al., 2014). Moreover, Notch 1 and Notch 2 appear to have opposite functions in embryonic brain tumors (Fan et al., 2004) and pancreatic ductal adenocarcinoma (PADC) (Hanlon et al., 2010; Mazur et al., 2010), adding another layer of complexity on the role of Notch signaling in tumor progression.

A relationship between cadherin and Notch signaling was suggested by the observation that Notch activity promotes AJ remodeling and cell morphogenesis via the regulation of E-cadherin spatial expression during *Drosophila* oogenesis (Grammont, 2007). E-cadherin, in normal transit-amplifying prostatic cells, can also affect Notch 1 signaling in a calcium-dependent manner. Reduced E-cadherin adhesion under conditions of low calcium promotes constitutive activation of Notch 1 and cell survival. Conversely, when these cells are grown in high calcium, Notch signaling is only active upon ligand-binding, and can be strengthened by E-cadherin-mediated adhesion (Dalrymple et al., 2005).

In endothelial cells, DLL4/Notch signaling cooperates with VEGF/VEGFR2 signaling to control the turnover of VE-cadherin and cell adhesion during vascular morphogenesis (Bentley et al., 2014) (Figure 5B). During angiogenesis, vascular sprouting from pre-existing vessels is regulated by the dynamic interaction of leading tip cells and stalk cells within each sprout. Cells encountering high Notch signals contain straight junctions and are static due to reduced junctional turnover of VE-cadherin, whereas cells encountering high VEGF exhibit serrated junctions and are highly motile, moving toward the leading tip. The impact of Notch on VE-cadherin junctions is also extended to endothelial barrier function (Mack et al., 2017; Polacheck et al., 2017) (Figure 5C). Using a microfabrication system to study endothelial responses to shear forces induced by fluid flow, Notch 1 was found to be associated with VE-cadherin under control conditions. However, under shear force Notch 1 was cleaved releasing its transmembrane domain (TMD), which recruited the receptor protein tyrosine phosphatase LAR, activated Rac1 signaling, and reinforced the AJs (Figure 5C). This adaptive response reinforced the barrier function of the endothelial monolayer to shear stress. Notably, this effect was independent of Notch transcriptional signaling but instead attributed to the physical interaction of VE-cadherin with the Notch TMD (Polacheck et al., 2017).

Bidirectional regulation between Notch2 and N-cadherin was reported in the context of chronic lymphocytic leukemia (CLL) (Mangolini et al., 2018). N-cadherin homotypic interactions between CLL cells and bone marrow-derived MSCs allow tumor cells to induce Notch2 activation in stromal cells. Active stromal Notch2, in turn, transcriptionally increases expression of N-cadherin, which surprisingly potentiates β-catenin stabilization and promotes Wnt signaling in CLL cells (Mangolini et al., 2018).

A close relationship between Notch and different cellular pools of β-catenin has also been suggested. Membrane-bound Notch physically interacts with dephosphorylated, active β-catenin, promoting its degradation (Hayward et al., 2005; Jin et al., 2009). This interaction is also observed in mouse embryonic stem cells where active β-catenin is downregulated by Notch in a ligand-independent manner. This Notch-dependent β-catenin degradation is surprisingly independent of the GSK3β/APC-mediated Destruction Complex and thus offers an alternative means to restrict the levels of active β-catenin and Wnt signaling in cells (Kwon et al., 2011). However, it is worth noting that ligand-induced Notch signaling in primary melanoma cells can upregulate β-catenin levels to promote tumor progression (Balint et al., 2005). Collectively, the data argue that Notch is a key regulator of β-catenin activity, but its effects are highly dependent on cellular context and subcellular localization. Conversely, β-catenin can also affect Notch signaling. Activation of Wnt by nuclear β-catenin can increase Notch signaling via the transcriptional activation of Jag1, leading to increased tumorigenesis (Rodilla et al., 2009; Kode et al., 2014).

Unlike Notch signaling, the HH pathway can promote both short-range and long-range signaling via HH ligands which are released by the transporter-like protein Dispatched (DISP1) (Burke et al., 1999; Zeng et al., 2001; Ma et al., 2002). HH ligands comprise sonic hedgehog (SHH), Indian hedgehog (IHH), and desert hedgehog (DHH) (Figure 5D). Other critical components in this pathway include the membrane receptors Patched (encoded by *PTCH1*) and Smoothened (encoded by *SMO*), the glioma-associated oncogene (GLI) TFs, and the adaptor protein suppressor of fused (SUFU). The function of HH signaling was initially revealed by studies on primary cilia, microtubule-enriched sensory organelles emanated from the plasma membrane of quiescent cells. In the absence of HH ligands, PTCH1 binds and prevents SMO translocation to the primary cilia (Gong et al., 2018). This inhibitory effect is released upon HH ligand binding to PTCH1, permitting cilia localization and activation of SMO. Activated SMO induces the release of GLI proteins from SUFU, which normally retains GLIs in the cytoplasm in the absence of HH ligands. Three GLI TFs (GLI1, GLI2, and GLI3) are the ultimate effectors of this pathway by regulating the expression of HH target genes Interestingly, while all three GLI proteins contain a C-terminal activation domain, GLI2 and GLI3 also possess repressor domains in their N-terminus and therefore can repress gene expression when their C-terminus is removed (Wu et al., 2017). GLI1 and GLI2 are widely considered as transcriptional activators (GLI^A^) in response to HH stimulation, whereas GLI3 mainly functions as a transcriptional repressor (GLI^R^) to inhibit HH target gene expression in the absence of HH ligands (Hui and Angers, 2011).

The oncogenic function of HH was initially suggested by the observation that loss of heterozygosity (LOH) and germline inactivating mutations in PTCH1 are associated with the Gorlin syndrome, a hereditary disorder that predisposes patients to basal cell carcinomas (BCCs), medulloblastomas, and rhabdomyosarcomas (Hahn et al., 1996; Johnson et al., 1996). Similarly, SUFU, the negative regulator of HH signaling, is also commonly mutated in Gorlin syndrome-related medulloblastomas (Taylor et al., 2002; Smith et al., 2014). In the setting of sporadic cancers, somatic deleterious mutations and deletions of PTCH1 and SUFU have been observed in a subset of BCCs (Teh et al., 2005; Jayaraman et al., 2014) and medulloblastomas (Raffel et al., 1997; Taylor et al., 2002). Activating mutations and overexpression of the oncogenic HH signaling components, such as SMO, are also linked to tumorigenesis in these cancers (Xie et al., 1998; Drummond et al., 2018; Tan et al., 2018). Enrichment of HH signaling is currently used as a biomarker in the clinic to guide targeted therapy for BCCs and to subcategorize medulloblastoma.

Similar to Notch, a bidirectional relationship exists between the HH pathway and AJs. Deletion of αE-catenin in neural progenitor cells causes disruption of apical junctions and loss of cell polarity, resulting in ectopic activation of HH signaling in the developing brain cortex (Lien et al., 2006) (Figure 5D). This, in turn, shortens the cell cycle and decreases cell death, leading to cortical hyperplasia and the formation of invasive tissue masses that resemble medulloblastoma and other brain tumors. In the zebrafish dorsal neural tube, impaired cell adhesion due to loss of functional N-cadherin also results in hyperactivation of HH signaling and hyperproliferation (Chalasani and Brewster, 2011) (Figure 5D). Activation of HH, in turn, promotes N-cadherin mediated AJ assembly as a negative feedback mechanism to restrict its own activity (Jarov et al., 2003; Fournier-Thibault et al., 2009; Chalasani and Brewster, 2011) (Figure 5D). Collectively, these studies indicated that AJs exert an inhibitory effect on HH signaling during neurulation.

Interestingly, Wnt/β-catenin signaling is critically involved in HH-mediated tumorigenesis (Yang et al., 2008). Moreover, the nuclear translocation of β-catenin induced by HH signaling can be suppressed by E-cadherin (Li et al., 2007; Inaguma et al., 2011). To circumvent this inhibitory effect of E-cadherin, HH signaling can either repress the expression or disrupt the junctional localization of E-cadherin. To repress E-cadherin expression HH promotes the expression of Snail or Maf TFs (Li et al., 2007; Inaguma et al., 2011; Lai et al., 2017). Additionally, mucin MUC5AC, a direct SHH target in pancreatic adenocarcinoma cells, is activated and its localization to intercellular junctions destabilizes E-cadherin adhesions (Inaguma et al., 2011). Overall, the data are consistent with a model whereby cadherin-mediated AJs suppress HH oncogenic signaling, whereas HH activation suppresses AJ function and promotes Wnt/β-catenin signaling.

The bilateral interactions between VE-cadherin and Notch modulated by hemodynamic stress as well as between N-cadherin and HH in the context of cell polarity have been nicely delineated in endothelial cells and central nervous system (CNS) precursor cells, respectively. However, our current understanding of the interplay between CCC, Notch, and HH signaling in cancer is far from complete. As Notch signaling can be tumor suppressing or promoting depending on cellular context, the role of CCCs in these different disease settings is unclear and warrants further investigation. Little is also known about the crosstalk between CCCs and HH signaling in human cancer despite strong evidence of negative feedback regulation between AJs and HH signaling during development, which is generally supported by cancer studies described in this review.

### Epithelial-mesenchymal transition and TGF-beta signaling

Metastasis involves a series of events from cell invasion and dissemination from the primary tumor mass to survival in circulation, extravasation and colonization of distant organs (Nieto et al., 2016). EMT, an important developmental process that regulates cell fate decisions and tissue specification, is often co-opted during metastasis to promote cell dissemination, followed by its reverse process mesenchymal-epithelial transition (MET) to re-establish the tumor at distant sites. Instead of a bimodal switch, EMT is now considered a dynamic and plastic program that includes a spectrum of EMT phenotypes involving intermediate partial and full EMTs (Nieto et al., 2016). Full EMT is characterized by loss of epithelial features accompanied by gain of mesenchymal phenotypes, endowing cells with invasive and migratory properties, whereas partial EMT states present varying degrees of epithelial and mesenchymal characteristics.

**FIGURE 6:**
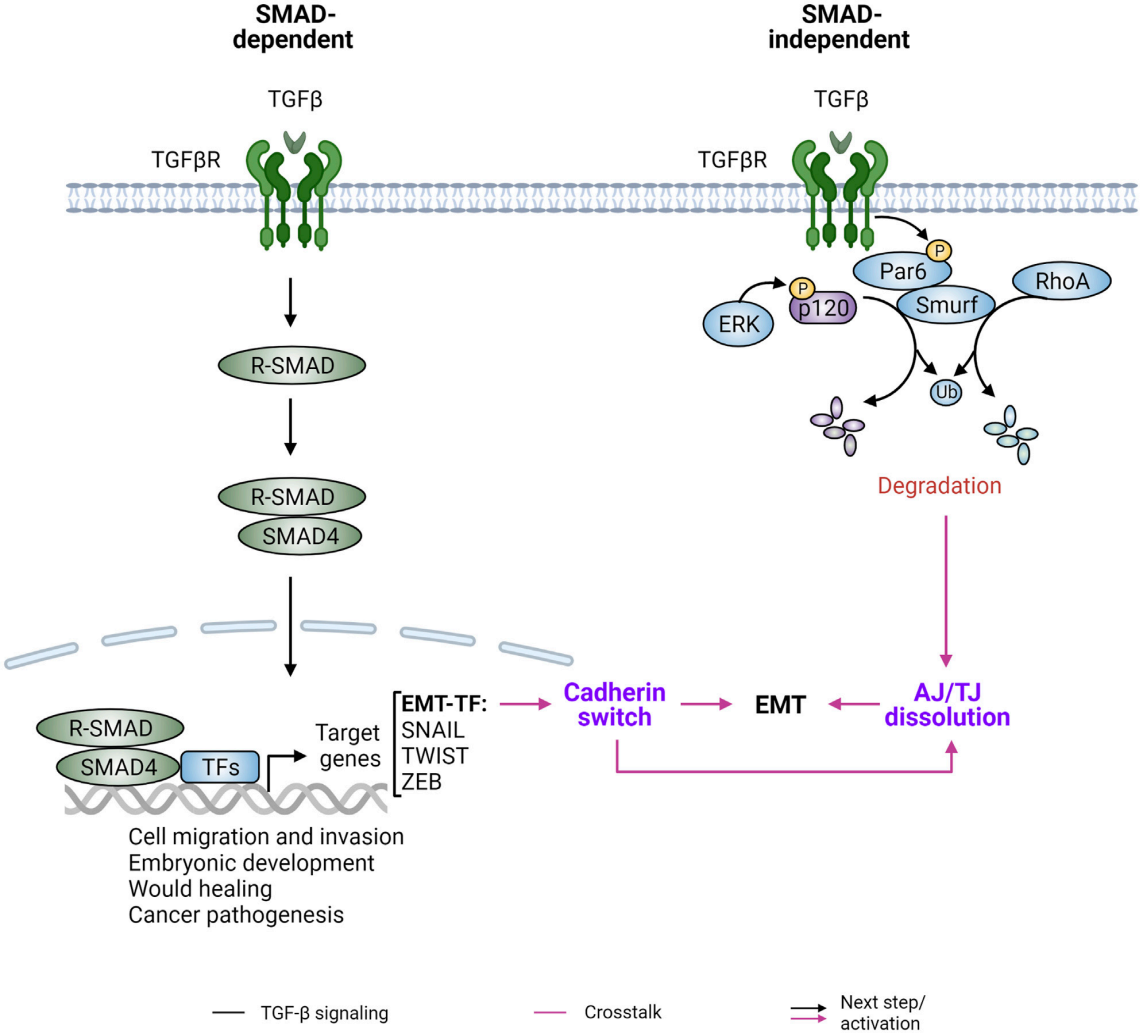
TGFβ signaling and cadherin/catenin plasticity. TGFβ signaling is a major inducer of epithelial-mesenchymal transition (EMT) and is mediated by SMAD-dependent and SMAD-independent downstream pathways. (Left) The canonical TGF-β signaling pathway uses Smad proteins to transduce its biological signals and effects. Upon TGFβ binding and the subsequent formation of the TGFβ-TGFβ receptor (TGFβRs) hetero-oligomeric signaling complex, receptor-activated SMADs (R-SMAD) are released from the TGFβ-TGFβR complex and form a heterotrimeric complex with SMAD4. The R-SMAD/SMAD4 complex then translocates into the nucleus and interacts with other transcription factors (TFs) and co-factors to drive expression of TGFβ target genes, such as the EMT master regulators SNAIL, TWIST, and ZEB. This leads to downregulation of E-cadherin and upregulation of N-cadherin, a process referred to as “cadherin switch.” (Right) The epithelial polarity protein partitioning-defective protein 6 (Par6) is one of the intracellular factors involved in SMAD-independent, non-canonical TGFβ signaling. Specifically, activated TGFβRII phosphorylates Par6, leading to the recruitment of the E3 ubiquitin ligase Smurf1 and localized degradation of RhoA and p120-catenin (p120) to affect downstream signaling. Figure created with BioRender.com.

A potent inducer of EMT is transforming growth factor beta (TGFβ), which functions in SMAD-dependent and independent manners (Lamouille et al., 2014) (Figure 6). The TGFβ family of ligands include TGFβs and bone morphogenetic proteins (BMPs), and they form homodimers or heterodimers to bind and activate TGFβ receptors. Activated receptors phosphorylate receptor-regulated SMADs (R-SMADs), which form a complex with SMAD4 to activate or repress gene expression following nuclear translocation. These nuclear SMAD complexes directly or indirectly promote the expression and transcriptional activity of EMT-TFs, including SNAIL1/2, ZEB1/2, and TWIST. These EMT-TFs function individually or collaboratively with each other or other TFs to repress expression of epithelial markers such as E-cadherin and increase the levels of mesenchymal markers such as N-cadherin, vimentin and fibronectin. This “cadherin switch” leads to altered cell-cell adhesion and potentiates EMT phenotypes. Independent of SMAD-mediated gene expression, TGFβ can also induce EMT phenotypes via the E3 ubiquitin ligase SMAD ubiquitination regulator factor 1 (SMURF1). SMURF1 is recruited to TGFβ receptors and targets localized RhoA for degradation, leading to dissociation of cortical actin (Ozdamar et al., 2005). Additionally, p120-catenin is mono-ubiquitinated by SMURF1 following TGFβ induced MAPK activation, leading to AJ and then tight junction (TJ) disassembly and ultimately lung metastasis of murine breast cancer (Wu et al., 2020) (Figure 6).

EMT can endow individual cells with decreased adhesion to the main tumor mass and with increased cell migration and invasion properties (Lamouille et al., 2014). This “identity crisis” is thought to allow tumor cells to disseminate, and to be critically involved in cancer metastasis. A vast body of evidence concurs that EMT promotes cell migration and invasion; however, its role in cancer metastasis remains under debate [reviewed in (Lamouille et al., 2014; Brabletz et al., 2018; Yang et al., 2020)]. Conditions at the leading edges of tumors favor a transient EMT that contributes to metastatic dissemination (Tsai et al., 2012; Tran et al., 2014; Puram et al., 2017). However, metastasis often involves groups of E-cadherin expressing cells that collectively migrate and invade tissues (Shamir et al., 2014; Westcott et al., 2015), and consistent with this, tumor cells with inhibited EMT still retain metastatic potential (Fischer et al., 2015; Zheng et al., 2015). For example, studies in mouse genetically engineered models (GEMM) suggested that EMT-mediated gene expression is dispensable for tumor cell invasion and metastasis, but rather promotes chemoresistance (Fischer et al., 2015; Zheng et al., 2015). Moreover, circulating tumor cell (CTC) clusters that contain a group of cells with intact epithelial junctions are more prevalent than single CTCs lacking E-cadherin expression in the blood of cancer patients (Friedl et al., 2012; Aceto et al., 2014), suggesting that retention of E-cadherin is common during key stages of metastatic spread.

The role of E-cadherin loss in cancer metastasis is also under debate, despite strong evidence that E-cadherin suppresses tumor cell migration, invasion, and anchorage-independent growth. Two recent studies used breast cancer mouse models to elucidate the role of E-cadherin in cancer metastasis (Padmanaban et al., 2019; Na et al., 2020). In one study, E-cadherin was conditionally knocked out in order to evaluate the role of E-cadherin expression in the metastasis cascade (Padmanaban et al., 2019), whereas the other used E-cadherin activating antibodies to elucidate the role of E-cadherin adhesive function in this process (Na et al., 2020). Both studies supported an inhibitory role of E-cadherin in cell invasion but showed opposite functions of E-cadherin in the formation of CTCs and gross metastasis. In the knockout study, E-cadherin loss decreased CTCs in the bloodstream and suppressed metastasis due to reduced cell proliferation and increased apoptosis (Padmanaban et al., 2019), while increased E-cadherin engagement in the second study resulted in suppression of cancer metastasis (Na et al., 2020). The discrepancies in E-cadherin effects could be related to cellular context and specifically the status of p53 and/or the apoptotic machinery. p120-catenin, α-catenin and E-cadherin are all thought to act as haploinsufficient tumor suppressors, whereby complete biallelic loss is only allowed under permissive conditions, like prior loss of p53 function (Shibata et al., 2007; Short et al., 2017). Conversely, epithelial cells expressing E-cadherin undergo cell death by anoikis upon anchorage independent conditions similar to those of CTCs in circulation, which may account for the increased apoptosis of CTCs and reduced metastasis of tumor cells treated with E-cadherin activating antibodies.

Our group reported that E-cadherin expression suppresses Rac1 and Src activation by mesenchymal cadherins resulting in reduced cell migration, invasion, and anchorage-independent growth (Yanagisawa and Anastasiadis, 2006; Soto et al., 2008). More recently we delineated two distinct E-cadherin containing complexes, one apical at the ZA containing PLEKHA7, and one basolateral lacking PLEKHA7 and characterized by increased Rac1 and Src activities (Kourtidis et al., 2015). Furthermore, the apical complex potently suppressed the pro-tumorigenic activity of the basolateral complex by recruiting the RNAi machinery to the ZA and regulating the translation of key pro-tumorigenic markers through a junction associated RNA induced silencing complex (RISC). The data argued that the tumor suppressive function of E-cadherin is specifically associated with the integrity of the apical ZA, and that in the absence of apical junctions, basolateral E-cadherin can promote tumor progression.

Despite contextual differences on the role of EMT and E-cadherin in cancer progression and metastasis, EMT remains an attractive therapeutic target to suppress metastasis and/or to overcome chemoresistance. While EMT does not only impact cell-cell adhesion, the switch in cadherin expression is a key part of the process. A deeper understanding of each tumor’s genomic and transcriptomic landscape, combined with the status of E-cadherin and the apical junctions, and elucidation of factors regulating dynamic EMT states may finally reconcile contextual differences and uncover the role of cadherin complexes in metastatic spread.

## Cadherin and catenin effects on the tumor immune microenvironment

### NF-κB and inflammation

The TME is crucial for tumor progression and comprises the ECM, stromal fibroblasts, endothelial cells, and tissue resident or infiltrating immune cells. These immune cells include lymphocytes and dendritic cells (DCs), which mediate adaptive immunity, and macrophages, natural killer (NK) cells, etc., which are involved in innate immunity. Through direct cell-cell interactions or communication via soluble factors, such as cytokines, the crosstalk between different cell types in the TME modulates biochemical pathways and cellular responses to suppress or promote tumor progression [reviewed in (Binnewies et al., 2018; Labani-Motlagh et al., 2020; Bejarano et al., 2021)].

**FIGURE 7:**
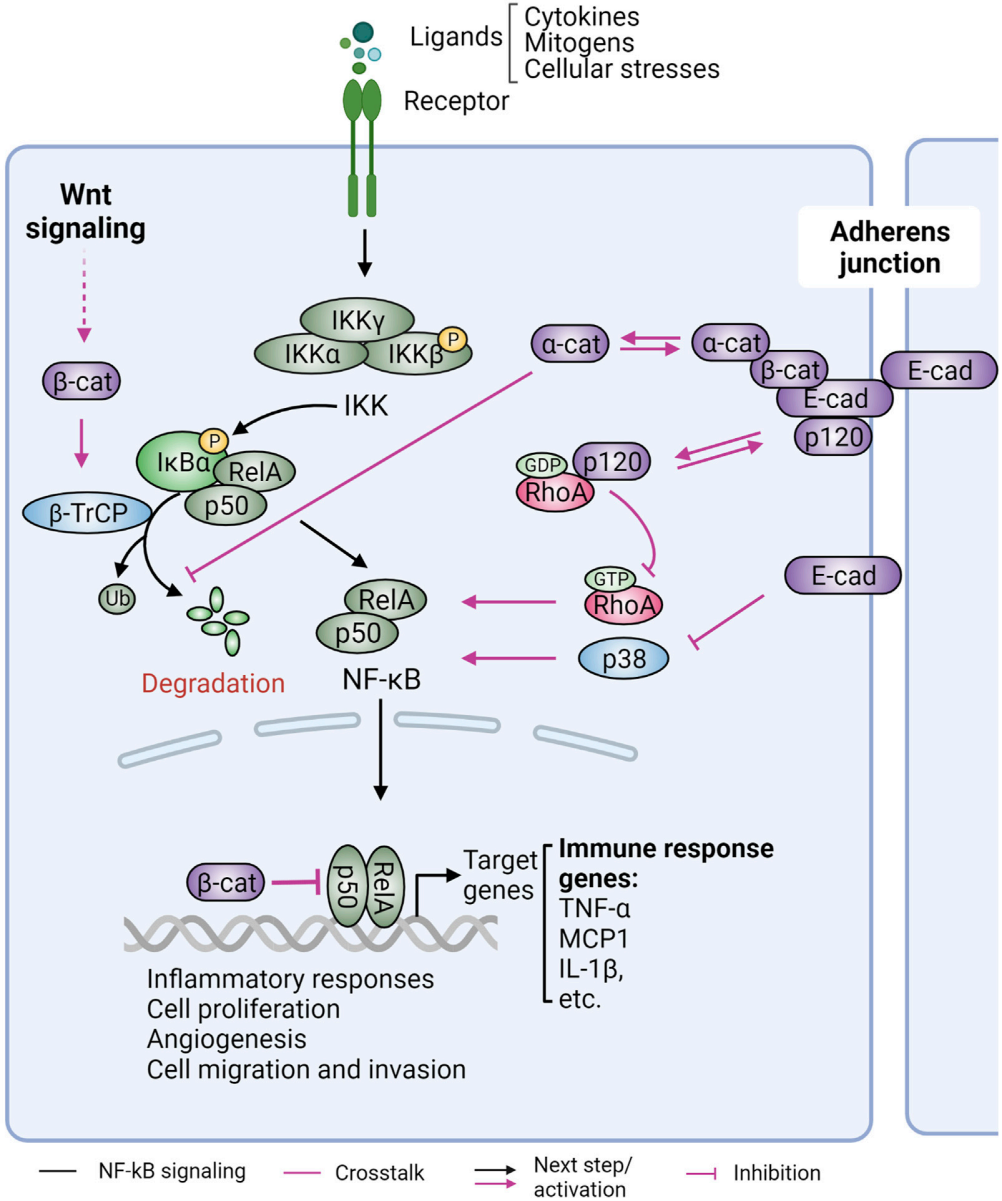
Role of cadherins and catenins in NF-κB signaling. Nuclear factor kappa B (NF-κB) signaling is a critical regulator of inflammation, an immune response that is linked to cancer formation and progression. Under normal conditions, NF-κB transcription factors (the most prominent dimer pair RelA-p50 shown) are retained in the cytoplasm by the inhibitor of κB (IκB). Various stimuli induce activation of the IκB kinase (IKK), which phosphorylates and inhibits IκB, resulting in the release and nuclear translocation of NF-κB and pro-inflammatory gene expression. Adherens junction proteins inhibit NF-κB signaling. E-cadherin (E-cad) loss in malignant melanoma cells blocks p38-mediated NF-kB activation. E-cadherin unbound p120-catenin (p120) functions as a guanine-nucleotide dissociation inhibitor to suppress RhoA activity, leading to NF-κB activation. Alpha-catenin (α-cat), independent of its binding to E-cadherin, interacts with IκBα to prevent IκBα ubiquitination and subsequent proteolytic degradation. Alpha-cat can also suppress NF-κB nuclear translocation. Beta-catenin (β-cat), on the other hand, can directly interact with NF-κB and repress its DNA-binding and transactivation activity. In response to canonical Wnt signaling, β-cat can stabilize β−TrCP1 mRNA. This results in elevated β−TrCP1 protein expression, which can increase NF-κB activation via downregulation of IκB. Figure created with BioRender.com.

The most studied role for cadherins and catenins in the TME is their effect on inflammatory responses via nuclear factor kappa B (NF-κB) signaling. NF-κB promotes inflammation, cell proliferation, angiogenesis, and cell migration and invasion and is frequently upregulated in cancer (Liu et al., 2017). In the absence of stimuli, NF-κB is sequestered in the cytoplasm by the inhibitor of κBs (IκBs) (Figure 7). In response to stimuli, such as cytokines, mitogens, or cellular stresses, IκB is phosphorylated and degraded, allowing nuclear translocation of NF-κB and expression of NF-κB target genes, driving inflammation.

Hermiston and Gordon initially reported that transgenic expression of a dominant negative N-cadherin in mouse small intestine epithelial cells results in inflammatory bowel disease (IBD) and epithelial dysplasia that leads to adenoma (Hermiston and Gordon, 1995). Loss of E-cadherin was then shown to induce activation of NF-κB signaling in malignant melanoma cells (Kuphal et al., 2004) (Figure 7). Furthermore, conditional knockout of p120-catenin in the mouse epidermis resulted in sustained inflammation, hyperproliferation, and skin cancer formation (Perez-Moreno et al., 2006; Perez-Moreno et al., 2008). Interestingly, the hyperplasia phenotype was linked to chronic inflammation and was independent of AJ instability, suggesting an involvement of soluble components. Indeed, a large array of pro-inflammatory cytokines (e.g., tumor necrosis factor alpha), interleukins (IL1β, IL6, IL13, and IL15), and chemokines [e.g., macrophage chemotactic protein (MCP1)] were substantially increased in *CTNND1* (p120-catenin) null epidermis and induced infiltration of immune cells. Mechanistically, cadherin-uncoupled p120-catenin inhibits guanine-nucleotide exchange to restrict RhoA activity (Anastasiadis et al., 2000). Loss of p120-catenin therefore results in the upregulation of RhoA-ROCK signaling, which leads to nuclear localization and activation of NF-κB (Figure 7). These inflammatory responses and the resulting tumor development were similarly observed when p120-catenin was knocked out in the intestine, squamous oral cavity, esophagus, forestomach, and a *TP53* null noninvasive breast cancer model (Smalley-Freed et al., 2010; Smalley-Freed et al., 2011; Stairs et al., 2011; Schackmann et al., 2013).

Alpha-catenin also has an anti-inflammatory role through the modulation of NF-κB signaling (Figure 7). Similar to p120-catenin ablation (Perez-Moreno et al., 2006), depletion of α-catenin results in NF-κB activation (Kobielak and Fuchs, 2006). In basal-like breast cancer cells, loss of α-catenin promotes NF-κB activation in an E-cadherin independent manner. A direct association of α-catenin with IκBα reportedly sustains IκBα stability and thus increases cytoplasmic retention of NF-kB (RelA) (Piao et al., 2014). β-catenin, on the other hand, exhibits a complicated relationship with NF-κB signaling by either suppressing or enhancing its activity in a context-dependent manner [reviewed in (Ma and Hottiger, 2016)]. In colon, liver, and breast cancer cells, a physical association of β-catenin with NF-κB is thought to decrease NF-κB’s DNA binding and transactivation activity (Deng et al., 2002; Du et al., 2009) (Figure 7). In prostate cancer cells, β-catenin also forms a transcription repressing complex to suppress NF-κB, resulting in downregulation of metastasis suppressor KAI1, a NFκB target gene (Kim et al., 2005) (Figure 7). Conversely, upregulation or increased stabilization of β-catenin by Wnt signaling resulted in NF-κB activation through upregulation of the β−TrCP E3 ubiquitin ligase, which mediates the degradation of NF-κB inhibitor protein IκB (Winston et al., 1999; Spiegelman et al., 2000; Noubissi et al., 2006) (Figure 7).

Overall, despite different degrees of involvement and underlying mechanisms, loss of cadherins (E-cadherin and N-cadherin) or catenins (p120-and α-catenin) results in NF-κB hyperactivation and production of pro-inflammatory signals that globally regulate the TME, induce immune infiltration and desmoplasia, and promote tumor progression.

### Immune regulation

In addition to their role in inflammation, increasing evidence supports a direct role of cadherin-catenin complexes in immune regulation. While not a focus of this review, compelling evidence exists supporting an immune suppressive role for Wnt/β-catenin signaling that also extends to resistance to treatment with immune checkpoint inhibitors (ICIs) (Ruiz de Galarreta et al., 2019; Du et al., 2020). Surprisingly, E-cadherin is expressed in certain immune cells and is critical for their function. In DCs, disruption of E-cadherin-mediated DC-DC adhesions triggers DC maturation via activation of β-catenin/TCF signaling. Intriguingly, different from the DCs that undergo the typical pathogen-induced maturation process, these DCs are linked to immune tolerance rather than immunity initiation (Jiang et al., 2007). Further, E-cadherin homophilic ligation links multiple myeloma cells and plasmacytoid DCs (pDCs), a subset of DCs known for their role in anti-virus innate immunity, and unexpectedly allows tumor cells to condition pDCs to promote tumor growth (Bi et al., 2018). Moreover, E-cadherin on epithelial cells can form heterotypic interactions with integrin αEβ7 on lymphocytes, recruiting these immune cells into epithelial tumors (Cepek et al., 1994; Le Floc’h et al., 2007). These studies highlight the largely unexplored roles of cadherins and catenins beyond the epithelium, and their potential in modulating the immune TME to regulate tumor development.

## Conclusion and perspectives

The diverse cellular functions regulated by cadherins and catenins highlight the importance of adhesion signaling in tumorigenesis and cancer progression. Extensive research has uncovered several molecular mechanisms by which cadherins/catenins regulate major oncogenic pathways involved in human cancer, and conversely, elucidated how oncogenic pathways regulate cadherin turnover and adhesive behaviors to promote cancer initiation and progression. The interplay between the two (cadherins/catenins and cancer pathways) is very dynamic and sensitive to their surrounding cells and the microenvironment in the tumor ecosystem.

This complexity stresses the importance of using physiological and disease relevant models to study cadherin/catenin biology and function in cancer. Human cancers are molecularly and cellularly complex comprised of sophisticated tissue architectures. Models that maintain this cellular diversity and overall architecture will be invaluable in elucidating the complex roles of CCCs in cancer. Significant questions remain related to the function of CCCs in cancer initiation, the TME, cancer progression, and metastasis. Addressing these questions will not only advance our understanding of signaling crosstalk between cadherin/catenin signaling and cancer driving events, but may also identify proper patient populations for given cancer therapies.

Notably, despite a wealth of information from TCGA, GWAS and functional studies regarding the expression, molecular alterations, and role of cadherins and catenins in particular cancer types, no directed treatment strategies are currently available to target cadherin/catenin dysfunction. Fortunately, several large-scale drug screening platforms have revealed potential lead compounds to target cadherin-dissociated β-catenin in Wnt signaling [reviewed in (Cui et al., 2018)]. Moreover, the introduction of novel gene therapy methods [reviewed in (Gregory and Copple, 2023)], epigenetic drugs, etc., may provide additional tools to target the multifaceted upregulation of oncogenic pathways induced by CCC dysfunction.
